# Association between antithrombotic therapy and mortality in patients hospitalized for COVID‑19

**DOI:** 10.1186/s12959-023-00572-6

**Published:** 2024-01-04

**Authors:** Xing Wang, Wuqian Chen, Jiulin Guo, Xingyu Qiu, Chao You, Lu Ma

**Affiliations:** 1 Department of Neurosurgery, West China Hospital, Sichuan University, Chengdu, Sichuan China; 2 Information Center, West China Hospital, Sichuan University, Chengdu, China

**Keywords:** Anticoagulants, COVID- 19, Mortality, Critical Illness, Antiplatelet

## Abstract

**Background:**

The prothrombotic state is a common abnormality in patients with coronavirus disease 2019 (COVID-19). However, there is controversy over the use of anticoagulants, especially oral anticoagulants (OAC) due to limited studies. We sought to evaluate the association between antithrombotic therapy on mortality and clinical outcomes in patients hospitalized for COVID-19 through propensity score matching (PSM) analysis.

**Methods:**

A retrospective cohort study was performed to include adult patients with COVID-19 in a university hospital. The primary outcome was in-hospital mortality. Secondary outcomes included intensive care unit (ICU) admission, mechanical ventilation, and acute kidney injury (AKI) during hospitalization. PSM was used as a powerful tool for matching patients’ baseline characteristics. Adjusted odds ratios (aOR) with 95% confidence intervals (CI) were calculated from the models.

**Results:**

Of 4,881 COVID-19 patients during the study period, 690 (14.1%) patients received antithrombotic therapy and 4,191 (85.9%) patients were under no antithrombotic therapy. After adjustment with multivariate regression analysis, patients receiving OAC, compared with those who did not receive any antithrombotic therapy, had significantly lower odds for in-hospital mortality (aOR: 0.46. 95% CI: 0.24 to 0.87; *P*= 0.017). PSM analysis observed similar results (aOR: 0.35. 95% CI: 0.19 to 0.61; *P*< 0.001). Moreover, in critically ill patients who received mechanical ventilation, antithrombotic treatment (aOR: 0.54. 95% CI: 0.32 to 0.89; *P*= 0.022) was associated with reduced risk of mortality.

**Conclusions:**

The application OACs was associated with reduced hospital mortality and mechanical ventilation requirement in COVID-19 patients. Besides, antithrombotic treatment was associated with a reduction in in-hospital mortality among critically ill COVID-19 patients who required mechanical ventilation.

**Supplementary Information:**

The online version contains supplementary material available at 10.1186/s12959-023-00572-6.

## Introduction

Severe acute respiratory syndrome coronavirus 2 (SARS-CoV-2) has infected over 184 million people and caused over 3.9 million deaths worldwide according to the latest report on 5 July by the WHO. The Corona Virus Disease 2019 (COVID-19) pandemic is contributing to increased risk of intensive care unit (ICU) admissions, and ultimately, increased mortality [1, 2]. Although the major clinical manifestations of COVID-19 involve respiratory symptoms, patients may develop prothrombotic complications that are associated with elevated mortality, with raised levels of both D-dimers and fibrinogen [3, 4]. The thrombotic abnormality often presents as pulmonary microvascular thrombosis, which can significantly impact the delivery of care and necessitate mechanical ventilation [5, 6]. Consequently, several antithrombotic approaches have been engaged in the management of this disease [7].

To date, several studies have investigated the effectiveness of antithrombotic agents including antiplatelets and anticoagulants in patients hospitalized with COVID-19, particularly for patients who require mechanical ventilation and intensive care [8]. Nonetheless, it remains controversial whether to prescribe antithrombotic drugs for these patients, as there are limited reports of randomized trials in this setting, especially for oral anticoagulants (OAC) [9, 10]. In general, the effects of OAC on clinical outcomes remain inconsistent. For example, one study found that thromboprophylaxis with rivaroxaban improved clinical outcomes in patients discharged after hospitalization due to COVID-19 [11]. While another study concluded that treatment with aspirin or apixaban did not reduce the rate of a composite clinical outcome among outpatients with COVID-19 [12]. Accordingly, a recent study did not demonstrate an impact of rivaroxaban on disease progression in adults with mild COVID-19, either [13]. However, most trials on the field have been criticized for the small sample size, which might lead to false negative results.

Recent research mostly focused on the administration of heparin, and the recommendations were substantially inconsistent. For example, current guidelines from the International Society on Thrombosis and Haemostasis (ISTH) recommend a prophylactic dose of low molecular weight heparin or unfractioned heparin to non-critically ill patients hospitalized for COVID-19 to reduce the incidence of thromboembolism and possibly death, but the panel did not recommend the use of antithrombotic therapy for critically ill, hospitalized patients [14]. However, these recommendations differed from another one which support the use of standard doses of heparin in critically ill patients [15]. Moreover, no recommendations have been made for OAC due to limited studies.

In this context, the objective of the present retrospective study was to investigate the effects of antithrombotic therapy on mortality and clinical outcomes in hospitalized patients with COVID-19.

## Methods

### Study design and data sources

We performed a retrospective, single-center, cohort study in West China Hospital, Sichuan University, for consecutively admitted patients with an electronic health record between December 2022 and February 2023. The institutional review board of the ethics committee of West China Hospital approved the study and granted a waiver of informed consent due to the minimal risk posed to patients.

### Patients

Inclusion criteria needed to be fulfilled: (1) patients admitted to West China Hospital of Sichuan University between December 2022 and February 2023 and diagnosed with SARS-CoV-2 infection; (2) patients aged 18 years or older; (3) confirmed cases of SARS-CoV-2 infection are identified by positive results of real-time reverse transcriptase-polymerase chain reaction, which involves amplification of virus-specific DNA sequences from lower respiratory aspirates or nasopharyngeal swabs, according to the criteria of World Health Organization. It should be noted that included patients were those who have confirmed COVID-19 infection with typical clinical manifestations, but COVID-19 might not be the reason for their admissions. In addition, we excluded (1) patients with acute illness including heart attack, organ bleeding, or patients with coagulation disorder, platelet dysfunction, or other situation (e.g. patients with an acute DVT or PE treated with heparin; severe thrombocytopenia) that may affect the use of antithrombotic therapy; (2) patients who received other forms of anticoagulants other than OAC.

### Demographics characteristics

Demographic data collected upon admission included age and gender, as well as the presence of chronic renal failure, hypertension, chronic obstructive pulmonary disease (COPD), diabetes mellitus, coronary heart disease (CHD), chronic liver diseases (CLD), presence of current smoking, and alcohol abuse.

### Exposure and outcomes

We investigated exposure to antithrombotic drugs use including antiplatelet, and oral anticoagulant on concerned outcomes in patients with COVID-19. Accordingly, the control patients were defined as those who were not taking of any anticoagulant or antiplatelet. The primary outcome was in-hospital mortality. Secondary outcomes included mechanical ventilation, ICU admission, and acute kidney injury (AKI) during hospitalization.

### Statistical analysis

All analyses were done with SPSS software (version 26; SPSS Inc) and R software (version 4.2.2; Foundation for Statistical Computing). Continuous variables with normal distribution were reported as means (with standard deviation [SD]), while those variables with skewed distribution were reported as median (with interquartile range). Categorical variables were reported as counts (frequencies). Mann-Whitney tests for variables with multiple categories and chi-square tests or Fisher’s exact test for binary variables. For continuous values with missing values, the random filling was imputed, and for categorical variables, all missing variables were coded as other. A 2-sided *P* value of < 0.05 was considered significant.

We analyzed categorical variables using univariable and multivariable logistic regression. From our experience and information from previous reports, age, sex, hypertension, diabetes mellitus, chronic renal failure, COPD, CHD, smoking, and alcohol abuse, were considered important confounders [16, 17]. Baseline variables and treatment related factors with *p*< 0.1 in the univariable regression were included in the multivariable logistic regression. In the multivariable analysis, adjusted odds ratios (aOR) with 95% confidence intervals (CI) were calculated.

Propensity scores were calculated using logistic regressions for each enrolled subject using the variables shown above [18, 19]. Covariates were selected based on the current scientific understanding of the variable’s potential association with the outcomes in COVID-19 patients. We performed a 1:1 matching without replacement using the nearest neighbor within the calipers method. The balance of covariates after matching was verified by examining the standardized mean difference (SMD); a difference of more than 0.1 is considered meaningful. Two analytic methods (before and after propensity score matching) were performed to obtain the aORs with 95% CI, and *p*< 0.05 was considered statistically significant.

## Results

**Fig. 1:**
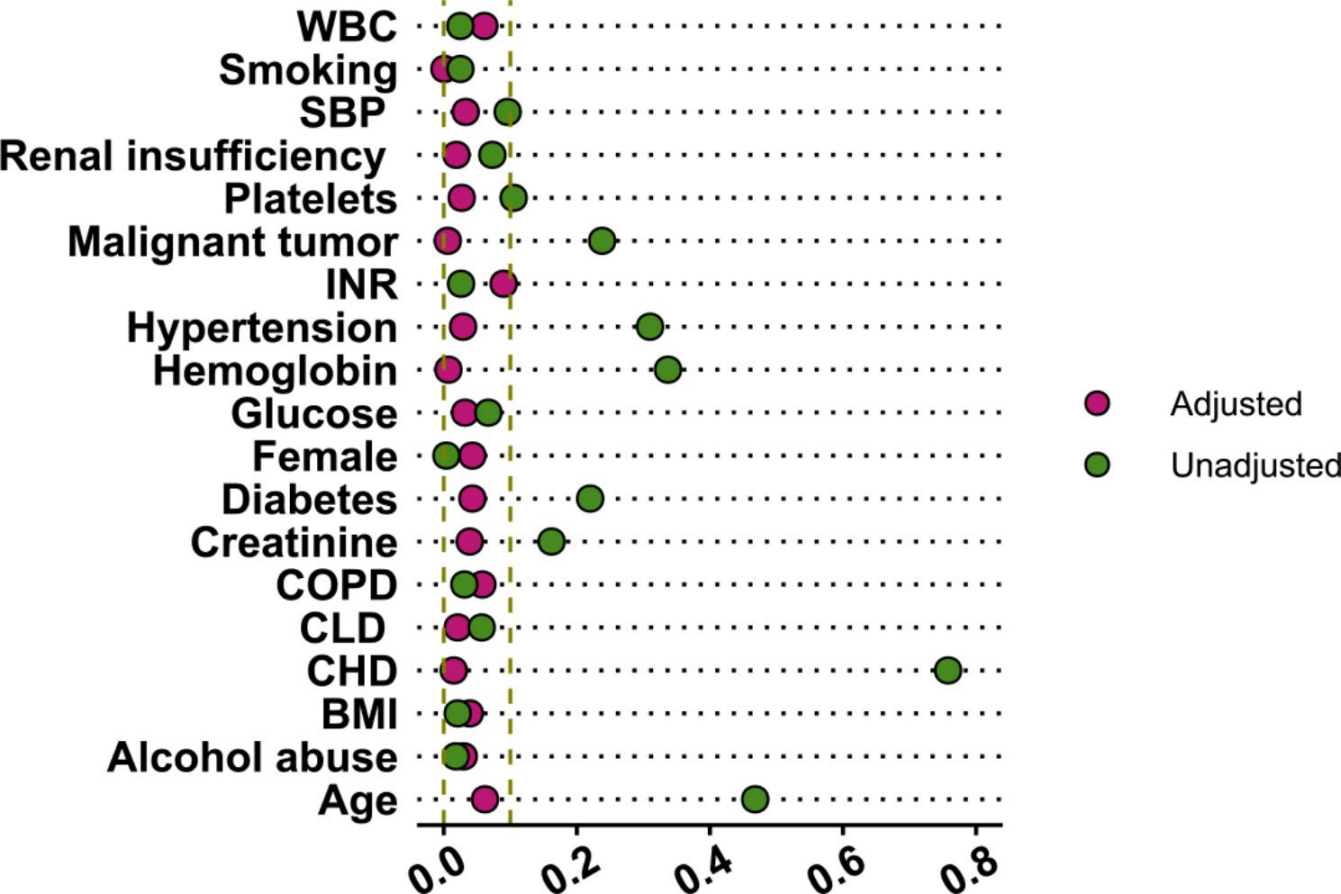
Propensity score matching effect evaluated by a dot plot. Red dots indicate the standard mean differences before matching; green dots indicate that after matching

During the study period, 4,881 COVID-19 patients were admitted, of whom 690 received antithrombotic therapy and 4,191 were did not receive any antithrombotic therapy. Patient characteristics at baseline are reported in Table 1. The mean age of patients included in the study was 64.8 (SD: 18.0). Patients in the cohort using antithrombotic agents (71.5 ± 15.0 years) were older than those receiving usual treatment (63.7 ± 18.2 years). Of note, the proportion of patients with hypertension (52.9% vs. 37.6%), coronary heart disease (42.6% vs. 11.2%), and diabetes (34.1% vs. 24.1%) are significantly higher in the antithrombosis group. While the proportion of patients with malignant tumors was significantly lower in patients treated with antithrombotic agents (6.5% vs. 13.6%). We did not notice a significant difference between the two groups regarding gender, body mass index, and several comorbidities (alcohol abuse, COPD, chronic renal failure, CLD). After matching, 690 cases of patients treated with antithrombotic agents were matched to 690 cases of patients who did not receive antithrombotic therapy (Fig. 1; Table S1). Overall, all covariates between patients under usual care and antithrombotic therapy remained comparable (SMD < 0.1).

**Table 1:** Patient characteristics by anticoagulant administration before PSM

Characteristics	Entire cohort	No antithrombotic therapy (n = 4191)	Antithrombotic therapy (n = 690)	*P*	SMD
Demographics					
Age, year; mean (SD)	64.79 (18.00)	63.69 (18.22)	71.49 (14.97)	< 0.001	0.468
Female, n (%)	1733 (35.5)	1487 (35.5)	246 (35.7)	0.965	0.004
Smoking, n (%)	987 (20.8)	843 (20.7)	144 (21.7)	0.583	0.025
Alcohol abuse, n (%)	737 (15.4)	637 (15.5)	100 (14.9)	0.716	0.018
Body mass index, kg/m^2^; mean (SD)	22.76 (3.55)	23.14 (3.84)	23.19 (3.70)	0.759	0.014
Medical history, n (%)					
Hypertension	1942 (39.8)	1577 (37.6)	365 (52.9)	< 0.001	0.310
Diabetes	1246 (25.5)	1011 (24.1)	235 (34.1)	< 0.001	0.220
COPD	498 (10.2)	422 (10.1)	76 (11.0)	0.489	0.031
CHD	762 (15.6)	468 (11.2)	294 (42.6)	< 0.001	0.758
Chronic renal failure	615 (12.6)	542 (12.9)	73 (10.6)	0.096	0.073
CLD	272 (5.6)	241 (5.8)	31 (4.5)	0.213	0.057
Malignant tumor	617 (12.6)	572 (13.6)	45 (6.5)	< 0.001	0.238
SBP, mmHg; mean (SD)	128.91 (20.82)	128.63 (20.79)	130.62 (20.96)	0.020	0.096
Laboratory events					
Hemoglobin, g/L; mean (SD)	113.02 (26.66)	111.85 (26.92)	120.40 (23.70)	< 0.001	0.337
Platelets, K/uL*	170 (116, 232)	169 (113.5, 232)	177 (128.25, 231.75)	0.017	0.105
WBC, K/uL*	6.5 (4.6, 9.59)	6.47 (4.56, 9.59)	6.67 (4.98, 6.67)	0.628	0.025
Creatinine, umol/L*	82 (64, 125)	81 (64, 126)	86 (67.75, 121.50)	0.001	0.162
Glucose, mmol/L; mean (SD)	7.66 (4.15)	7.63 (4.20)	7.89 (3.85)	0.134	0.067
INR; mean (SD)	1.12 (0.38)	1.12 (0.38)	1.11 (0.36)	0.573	0.026

WBC: white blood cell; INR: international normalized ratio. Hemoglobin data were missing in 282 (5.78%) of 4,881 patients; platelets data were missing in 284 (5.81%) of 4,881 patients; WBC data were missing in 282 (5.78%) of 4,881 patients; creatinine data were missing in 274 (5.61%) of 4,881 patients; glucose data were missing in 290 (5.94%) of 4,881 patients; INR data were missing in 820 (16.8%) of 4,881 patients; and BMI data were missing in 870 (17.8%) of 4,881 patients

^*^ items are presented as median and quartiles

Table 2 exhibits unadjusted and adjusted associations between antithrombotic therapy and in-hospital outcomes. After adjustment with multivariate regression analysis, patients receiving antithrombotic therapy, compared with those receiving usual care, had significantly lower odds for in-hospital mortality (aOR: 0.67. 95% CI: 0.46 to 0.98; *P*= 0.038; Table S2), and ICU admission (aOR: 0.41. 95% CI: 0.25 to 0.69; *P*= 0.001). In propensity score matching analysis, similar results were observed. Patients treated with antithrombotic agents were associated with reduced risk of all-cause mortality in hospital (aOR: 0.55. 95% CI: 0.39 to 0.77; *P*= 0.001), application of mechanical ventilation (aOR: 0.65. 95% CI: 0.50 to 0.83; *P*= 0.001), and ICU admission (aOR: 0.51. 95% CI: 0.31 to 0.83; *P*= 0.009).

**Table 2:** In-hospital complications stratified by treatment regimen (antithrombotic therapy vs. no antithrombotic therapy)

Outcomes	Unadjusted		Multivariable Regression Adjustment		Propensity Score Matching Adjustment	
	OR (95% CI)	*p*	OR (95% CI)	*p*	OR (95% CI)	*p*
In-hospital mortality	0.76 (0.57, 1.01)	0.066	0.67 (0.46, 0.98)	0.038	0.55 (0.39, 0.77)	0.001
Mechanical ventilation	0.76 (0.62, 0.93)	0.009	0.78 (0.59, 1.01)	0.062	0.65 (0.50, 0.83)	0.001
ICU administration	0.48 (0.31, 0.71)	< 0.001	0.41 (0.25, 0.69)	0.001	0.51 (0.31, 0.83)	0.009
AKI	0.57 (0.32, 0.94)	0.050	0.49 (0.24, 1.02)	0.056	0.71 (0.36, 1.38)	0.398

We further assessed the in-hospital complications risk of oral anticoagulants or antiplatelets respectively (Table 3). A total pf 419 patients were treated with antiplatelet only; 271 patients were treated with OAC with/without antiplatelet. The most frequently used OAC was rivaroxaban (239/271); followed by dabigatran (26/271), and warfarin (6/271). Among patients treated rivaroxaban, 56.7% of them were treated with low-dose therapies (≤ 10 mg once daily); 43.3% of them were treated with high-dose therapies (> 10 mg once daily). Among patients treated with dabigatran, 31.0% were treated with 110 mg, once daily; 69.0% were treated with 110 mg, twice daily. Among patients treated with warfarin, the treatment regimen was more individualized, with doses ranging from 0.625 mg once daily to 3.75 mg once daily.

**Table 3:** Unadjusted and adjusted associations between different treatments and mortality

Outcomes	Treatment regimen	Events, n (%)	Unadjusted OR	P	Multivariable Regression adjusted OR	P	Propensity Score Matching adjusted OR	P
In-hospital mortality	No anticoagulant and no antiplatelet	473 (11.3)	1 [Reference]		1 [Reference]		1 [Reference]	
	Antiplatelet	44 (10.5)	0.92 (0.67, 1.28)	0.627	0.83 (0.53, 1.30)	0.413	0.63 (0.42, 0.94)	0.032
	OAC with/without antiplatelet	17 (6.3)	0.53 (0.32, 0.87)	0.012	0.46 (0.24, 0.87)	0.017	0.35 (0.19, 0.61)	< 0.001
Mechanical ventilation	No anticoagulant and no antiplatelet	975 (23.3)	1 [Reference]		1 [Reference]		1 [Reference]	
	Antiplatelet	82 (19.6)	0.80 (0.62, 1.03)	0.087	0.89 (0.64, 1.23)	0.470	0.72 (0.52, 0.99)	0.047
	OAC with/without antiplatelet	47 (17.3)	0.69 (0.50, 0.96)	0.025	0.62 (0.41, 0.95)	0.028	0.49 (0.33, 0.74)	0.001
ICU administration	No anticoagulant and no antiplatelet	315 (7.5)	1 [Reference]		1 [Reference]		1 [Reference]	
	Antiplatelet	13 (3.1)	0.39 (0.22, 0.69)	0.001	0.35 (0.18, 0.71)	0.003	0.33 (0.17, 0.62)	0.001
	OAC with/without antiplatelet	13 (4.8)	0.62 (0.35, 1.10)	0.100	0.51 (0.24, 1.06)	0.070	0.60 (0.29, 1.21)	0.215
AKI	No anticoagulant and no antiplatelet	157 (3.7)	1 [Reference]		1 [Reference]		1 [Reference]	
	Antiplatelet	6 (1.4)	0.37 (0.16, 0.85)	0.019	0.38 (0.14, 1.06)	0.063	0.29 (0.11, 0.69)	0.010
	OAC with/without antiplatelet	9 (3.3)	0.88 (0.45, 1.75)	0.720	0.68 (0.24, 1.88)	0.455	1.30 (0.48, 3.67)	0.800

Compared with patients who received neither oral anticoagulant nor antiplatelet therapy, those taking OAC with/without antiplatelet had significantly lower odds for in-hospital mortality (aOR: 0.46. 95% CI: 0.24 to 0.87; *P*= 0.017), application of mechanical ventilation (aOR: 0.62. 95% CI: 0.41 to 0.95; *P*= 0.028). While intervention with antiplatelet alone was associated with reduced incidence of ICU admission (aOR: 0.35. 95% CI: 0.18 to 0.71; *P*= 0.003). Those results were validated in propensity score matching analyses. Patients taking OAC with/without antiplatelet were associated with reduced risk of all-cause mortality in the hospital (aOR: 0.35. 95% CI: 0.19 to 0.61; *P*< 0.001; Tables S3-S4).

**Fig. 2:**
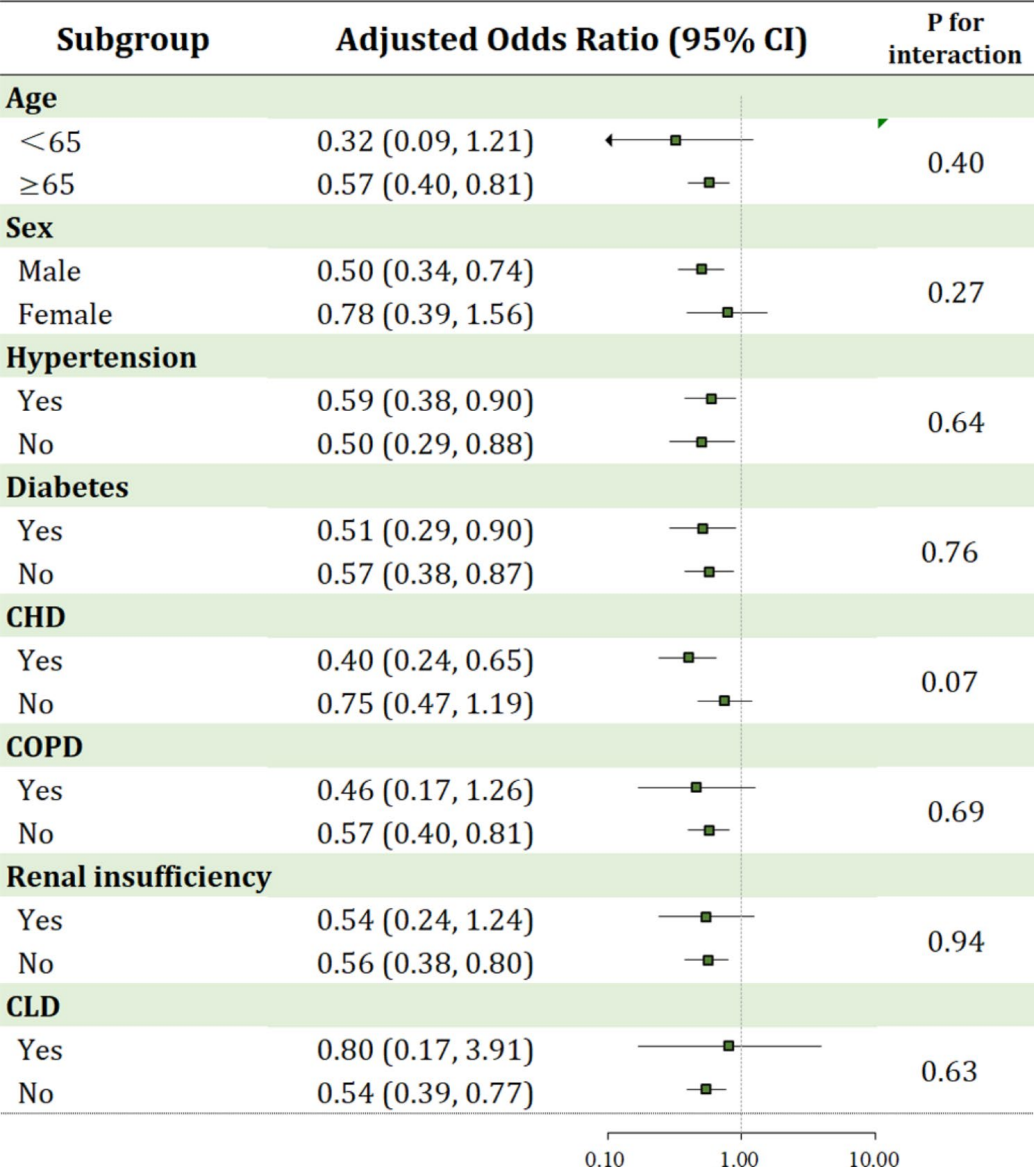
Subgroup analyses of antithrombotic therapy for in-hospital mortality. The analyses were performed using the matching data

**Fig. 3:**
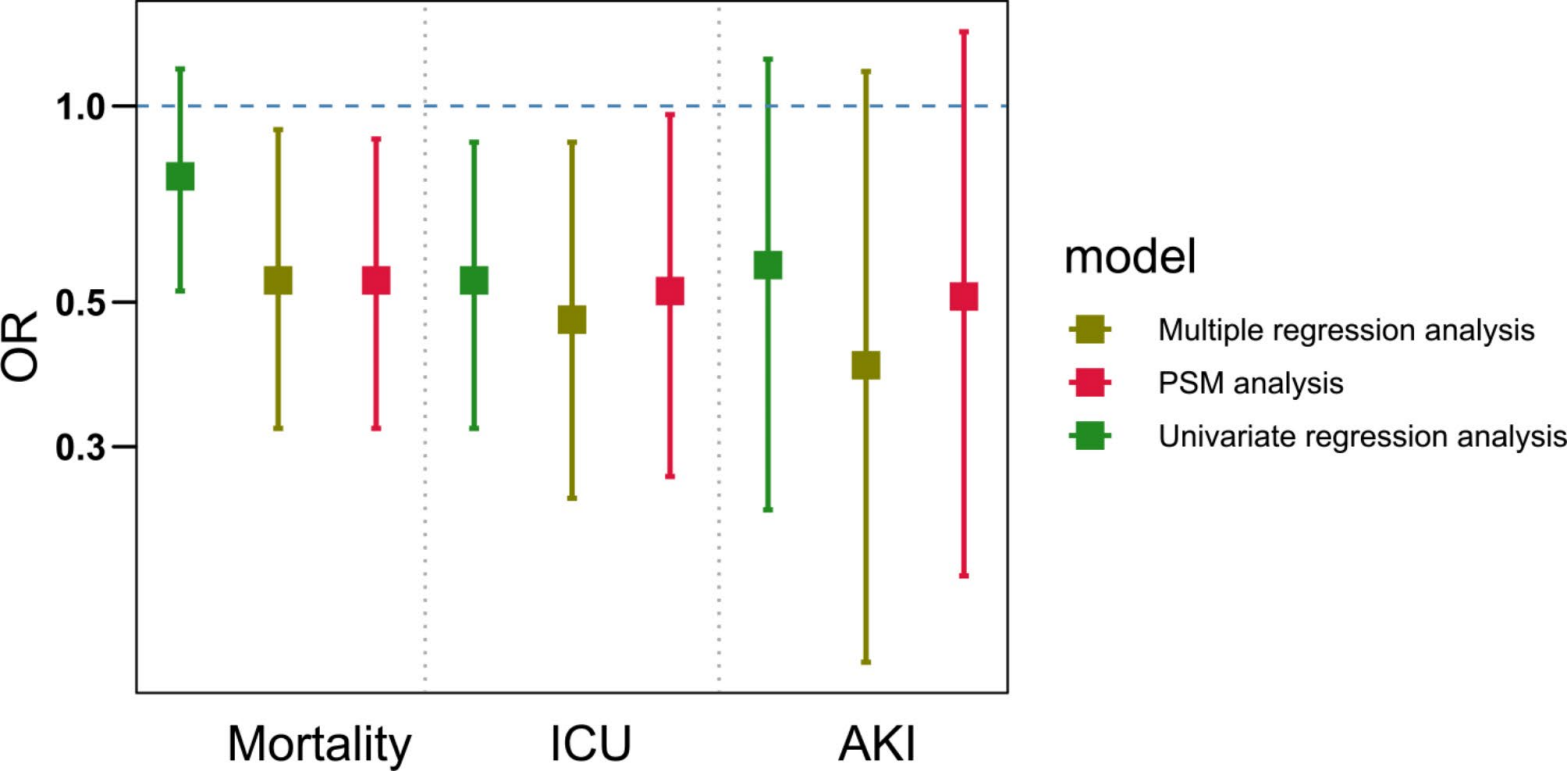
Comparison of antithrombotic therapy vs. no antithrombotic therapy on mortality, ICU admission, and AKI in COVID-19 patients receiving mechanical ventilation

Subgroup analyses were performed to assess the interactions between different statuses on in-hospital mortality using matching data (Fig. 2). We did not discover any difference in mortality between patients receiving antithrombotic therapy and those who did not receive antithrombotic therapy regarding different baseline characteristics. We also conducted subgroup analysis in patients on mechanical ventilation. The results revealed that patients in antithrombotic group had significantly lower odds for in-hospital mortality (aOR: 0.54. 95% CI: 0.32 to 0.92; *P*= 0.024) compared with patients who received neither oral anticoagulant nor antiplatelet therapy. Similar results were observed in the analysis of the PSM cohort (Fig. 3). Moreover, our analysis suggested that in COVID-19 patients receiving mechanical ventilation, those treated with OAC with/without antiplatelet (aOR: 0.22. 95% CI: 0.08 to 0.60; *P*= 0.003; Table S5) were associated with reduced incidence of in-hospital mortality compared with non-antithrombotic therapy.

## Discussion

### Main findings

This retrospective cohort study was performed to determine the impact of antithrombotic therapy on clinical outcomes in hospitalized patients with COVID-19. After adjustment with multivariate regression analysis and propensity score matching analysis, we found that patients receiving antithrombotic therapy, particular OACs during hospitalization have significantly lowered odds of in-hospital complications and death. Although causality has not been proven, this provides further impetus to administer anticoagulant therapy to hospitalized patients with COVID-19.

Of this cohort analyzed from a single university hospital between December 2022 and February 2023, 14.14% of patients received antithrombosis administration. Patients on antithrombotic therapy had lower in-hospital mortality rates than the control group. In addition, antithrombotic therapy during hospitalization for COVID-19 was associated with a lower risk of ICU admission and mechanical ventilation. The findings of our study were consistent with previous studies that antithrombotic therapy improves COVID-19 outcomes. For example, Adler, L. et al. conducted a retrospective cohort consisting of 6,884 patients diagnosed with COVID-19 in skilled nursing facilities and reported that application of antithrombotic agents was associated with a lower rate of 30-day mortality compared with those not treated with these medications [20]. Handy et al., reported in a large atrial fibrillation cohort, in COVID-19 positive patients, pre-existing antithrombotic use was associated with lower odds of COVID-19 death [21]. In addition, patients hospitalized for COVID-19 receiving anticoagulant therapy were reported lower ICU admission rates [22].

It’s worth noting that in a subgroup analysis of patients on mechanical ventilation in this cohort, OAC use was associated with reduced mortality (OR: 0.22, 95% CI: 0.08 to 0.60). The results remained statistically significant after propensity score matching adjustment, suggesting the reliability of the findings. This provides the incentive to improve antithrombotic therapy coverage in individuals hospitalized for COVID-19 received mechanical ventilation. However, it should be noted that patients in the present study received differed dosage of antithrombotic agent. Recently, Paranjpe, I. et al. found that treatment-dose anticoagulant therapy is associated with reduced in-hospital mortality in patients requiring mechanical ventilation [23]. Further studies are warranted to explore the comparative effectiveness of prophylactic versus therapeutic doses of anticoagulants in these patients.

Accordingly, the present analysis suggested that certain subgroup of patients might benefit more from antithrombotic therapy; however, the *p* value for interaction in each comparison did not reach statistically significance. Therefore, sufficiently powered randomized controlled trials are needed to assess whether a causal relationship exists between anticoagulant use and reduced mortality in certain type of patients with COVID-19.

### Clinical implications

The guidelines from several international and American societies recommend prophylactic doses of anticoagulation to all hospitalized COVID-19 patients unless contraindications exist [8, 14, 24]. The recommended anticoagulant drugs are mostly heparin, including low molecular weight heparin and unfractionated heparin. Studies suggest that heparin has beneficial effects in preventing COVID-19-related thrombosis and reducing mortality [3, 25]. In this study, we focus on direct OAC and antiplatelet therapy and found that they are all associated with better outcomes in COVID-19 patients. Previous randomized control trials showed that for symptomatic outpatients with COVID-19, aspirin or apixaban did not improve the combined clinical outcome as compared with placebo [12, 26]. However, in a retrospective observational cohort study, patients hospitalized with COVID-19 received combined anticoagulant and antiplatelet therapy was associated with a better outcome in comparison to prophylactic anticoagulation alone [27], suggesting the therapeutic effect of antiplatelet therapy on patients with COVID-19. The prothrombotic and hypercoagulable state has been observed in hospitalized individuals with SARS-CoV-2 infection [17], and aspirin has multiple roles of anti-inflammatory, antithrombotic, and antiviral properties as an adjunctive drug in COVID-19 treatment, so it would also be effective in novel coronavirus variants [28]. We did not directly study thrombotic events and hemorrhagic complications, but it is noteworthy that higher doses of anticoagulants result in increased rates of bleeding [16]. The potential benefits of antithrombotic therapy need to be weighed against the risk of bleeding and thus should be individualized.

### Strengths and limitations

This study has several notable strengths. We compared different anticoagulation strategies, including antiplatelet agents and OAC for hospitalized patients with COVID-19, and thus filled a gap in current research by exploring their impact on in-hospital mortality and complications. Subgroup analysis was conducted for patients undergoing mechanical ventilation, and the results suggested a potential benefit of OAC in reducing mortality in critically ill patients. Propensity score matching was applied as a powerful tool for matching patients’ baseline characteristics, thus yielding well-balanced covariates in each cohort. The consistency of the results between the conventional multivariate analysis and the propensity score matching analysis confirmed the reliability of the findings.

The study does have limitations. First, the retrospective and observational designs have unmeasured and residual confounders; therefore, no causality can account for the association between the antithrombotic therapy and outcomes. Second, we lack metrics to perform subgroup analyses of the dose, duration of antithrombotic therapy, or the time to initiation of therapy. We did not perform an analysis of prehospitalization antithrombotic therapies, so our analyses cannot rule out differences in COVID-19 results due to the use of additional anticoagulation regimens or other medications.

## Conclusion

In conclusion, the findings from this large cohort study of consecutive hospitalized patients with COVID-19 indicated that OACs were associated with reduced hospital mortality and mechanical ventilation requirement. In addition, the application of antithrombotic agents was associated with a reduction in in-hospital mortality among critically ill COVID-19 patients who required mechanical ventilation.

### Electronic supplementary material

Below is the link to the electronic supplementary material.

Supplementary Material 1

Supplementary Material 2

## Data Availability

The data generated or analyzed during the current study are available from the corresponding author on reasonable request.

## Abbreviations

COVID-19: coronavirus disease 2019
OAC: oral anticoagulants
PSM: propensity score matching
ICU: intensive care unit
aOR: adjusted odds ratios
CI: confidence intervals
SARS-CoV-2: severe acute respiratory syndrome coronavirus 2
ISTH: International Society on Thrombosis and Haemostasis
AKI: acute kidney injury
SMD: standardized mean difference
