# Supplementary material for: Association between antithrombotic therapy and mortality in patients hospitalized for COVID‑19
Source: Thromb J. 2024 Jan 4;22:5. doi: 10.1186/s12959-023-00572-6 (PMC10768353; doi:10.1186/s12959-023-00572-6)
Supplement: Supplementary file 2 — Supplementary Material 2 [file 12959_2023_572_MOESM2_ESM.docx]

**Table S1. Patient characteristics after propensity score matching by treatment regimen (antithrombotic therapy vs. no antithrombotic therapy)**

| **Characteristics** | **Non-antithrombotic (n=690)** | **Anticoagulant or antiplatelet (n=690)** | **p value** | **SMD** |
| --- | --- | --- | --- | --- |
| Demographics |  |  |  |  |
| Age, year, mean (SD) | 72.45 (15.86) | 71.49 (14.97) | 0.247 | 0.062 |
| Female, n (%) | 232 (33.6) | 246 (35.7) | 0.462 | 0.043 |
| Smoking, n (%) | 144 (21.7) | 144 (21.7) | 1.000 | 0.001 |
| Alcohol abuse, n (%) | 93 (13.8) | 100 (14.9) | 0.641 | 0.030 |
| Body mass index, kg/m^2^; mean (SD) | 23.50 (3.90) | 23.19 (3.70) | 0.175 | 0.081 |
| Medical history, n (%) |  |  |  |  |
| Hypertension | 375 (54.3) | 365 (52.9) | 0.627 | 0.029 |
| Diabetes | 221 (32.0) | 235 (34.1) | 0.457 | 0.043 |
| COPD | 89 (12.9) | 76 (11.0) | 0.319 | 0.058 |
| CHD | 299 (43.3) | 294 (42.6) | 0.828 | 0.015 |
| Chronic renal failure | 77 (11.2) | 73 (10.6) | 0.795 | 0.019 |
| CLD | 34 (4.9) | 31 (4.5) | 0.799 | 0.021 |
| Malignant cancer | 44 (6.4) | 45 (6.5) | 1.000 | 0.006 |
| SBP, mmHg; mean (SD) | 131.26 (20.84) | 130.62 (20.96) | 0.575 | 0.030 |
| Laboratory events |  |  |  |  |
| Hemoglobin, g/L; mean (SD) | 118.18 (24.74) | 120.40 (23.70) | 0.102 | 0.937 |
| Platelets, K/uL^*^ | 178 (126, 234) | 177 (128.25, 231.75) | 0.884 | 0.008 |
| WBC, K/uL^*^ | 7.02 (4.93, 10.01) | 6.67 (4.98, 6.67) | 0.728 | 0.020 |
| Creatinine, umol/L^*^ | 83 (66, 119) | 86 (67.75, 121.50) | 0.738 | 0.019 |
| Glucose, mmol/L | 8.09 (4.21) | 7.89 (3.85) | 0.387 | 0.048 |
| INR; mean (SD) | 1.11 (0.32) | 1.11 (0.36) | 0.910 | 0.007 |

COPD: chronic obstructive pulmonary disease; CLD: chronic liver diseases; CHD: coronary heart disease; SBP: systolic blood pressure; SMD: standardized mean difference; SD: standard deviation; WBC: white blood cell; INR: international normalized ratio

^*^ items are presented as median and quartiles.

**Table S2. Unadjusted and risk-adjusted analysis for mortality (antithrombotic therapy vs. no antithrombotic therapy).**

| Characteristics | Unadjusted | | Multivariable Regression Adjustment | |
| --- | --- | --- | --- | --- |
|  | OR (95% CI) | p value | OR (95% CI) | p value |
| Demographics |  |  |  |  |
| Age | 1.06 (1.05, 1.07) | <0.001 | **1.07 (1.06, 1.08)** | **<0.001** |
| Female | 0.51 (0.41, 0.63) | <0.001 | **0.54 (0.39, 0.74)** | **<0.001** |
| Smoking | 1.16 (0.93, 1.44) | 0.203 |  |  |
| Alcohol abuse | 0.90 (0.69, 1.17) | 0.487 |  |  |
| Body mass index | 0.96 (0.94, 0.99) | 0.011 | 1.00 (0.97, 1.04) | 0.819 |
| Medical history |  |  |  |  |
| Hypertension | 1.83 (1.53, 2.19) | <0.001 | 1.17 (0.87, 1.56) | 0.293 |
| Diabetes | 1.59 (1.31, 1.93) | <0.001 | 0.79 (0.57, 1.10) | 0.165 |
| COPD | 1.57 (1.20, 2.03) | 0.001 | 0.79 (0.53, 1.16) | 0.224 |
| CHD | 1.51 (1.20, 1.88) | <0.001 | 0.89 (0.63, 1.25) | 0.491 |
| Chronic renal failure | 2.20 (1.75, 2.74) | <0.001 | **1.56 (1.05, 2.31)** | **0.029** |
| CLD | 0.93 (0.61, 1.37) | 0.801 |  |  |
| Malignant tumor | 0.81 (0.60, 1.07) | 0.168 |  |  |
| SBP | 1.01 (1.00, 1.01) | 0.003 | 1.00 (0.99, 1.01) | 0.548 |
| Laboratory events |  |  |  |  |
| Hemoglobin | 0.99 (0.98, 1.00) | <0.001 | 1.00 (0.99, 1.01) | 0.161 |
| Platelets | 0.98 (0.98, 0.99) | <0.001 | 0.99 (0.98, 0.99) | **<0.001** |
| WBC | 1.06 (1.04, 1.08) | <0.001 | 1.03 (1.01, 1.04) | **<0.001** |
| Creatinine | 1.00 (1.00, 1.00) | <0.001 | 1.00 (0.99, 1.01) | 0.219 |
| Glucose | 1.12 (1.10, 1.14) | <0.001 | **1.11 (1.08, 1.14)** | **<0.001** |
| INR | 2.56 (2.03, 3.25) | <0.001 | **2.10 (1.62, 2.73)** | **<0.001** |
| Anticoagulant or antiplatelet | 0.76 (0.57, 1.01) | 0.066 | **0.67 (0.46, 0.98)** | **0.038** |

COPD: chronic obstructive pulmonary disease; CLD: chronic liver diseases; CHD: coronary heart disease; SBP: systolic blood pressure; SD: standard deviation; WBC: white blood cell; INR: international normalized ratio

| Characteristics | Before matching | | | After matching | | |
| --- | --- | --- | --- | --- | --- | --- |
|  | Non-antithrombotic (n=4191) | Antiplatelet (n=419) | SMD | Non-antithrombotic (n=419) | Antiplatelet (n=419) | SMD |
| Demographics |  |  |  |  |  |  |
| Age | 63.69 (18.22) | 71.00 (14.66) | 0.442 | 72.11 (15.30) | 71.00 (14.66) | 0.074 |
| Female | 1487 (35.5) | 125 (29.8) | 0.121 | 133 (31.7) | 125 (29.8) | 0.041 |
| Smoking | 955 (22.8) | 121 (28.8) | 0.151 | 112 (26.7) | 121 (28.8) | 0.048 |
| Alcohol abuse | 722 (17.2) | 80 (19.1) | 0.063 | 72 (17.2) | 80 (19.1) | 0.061 |
| Body mass index | 22.75 (3.57) | 22.92 (3.38) | 0.049 | 22.85 (3.60) | 22.92 (3.38) | 0.021 |
| Medical history |  |  |  |  |  |  |
| Hypertension | 1577 (37.6) | 244 (58.2) | 0.422 | 256 (61.1) | 244 (58.2) | 0.058 |
| Diabetes | 1011 (24.1) | 155 (37.0) | 0.282 | 147 (35.1) | 155 (37.0) | 0.040 |
| COPD | 422 (10.1) | 33 (7.9) | 0.077 | 31 (7.4) | 33 (7.9) | 0.018 |
| CHD | 468 (11.2) | 208 (49.6) | 0.921 | 209 (49.9) | 208 (49.6) | 0.005 |
| Chronic renal failure | 542 (12.9) | 48 (11.5) | 0.045 | 54 (12.9) | 48 (11.5) | 0.044 |
| CLD | 241 (5.8) | 19 (4.5) | 0.055 | 24 (5.7) | 19 (4.5) | 0.054 |
| Malignant cancer | 572 (13.6) | 21 (5.0) | 0.300 | 22 (5.3) | 21 (5.0) | 0.011 |
| SBP | 128.52 (20.73) | 133.04 (21.38) | 0.215 | 131.12 (20.98) | 133.04 (21.38) | 0.091 |
| Laboratory events |  |  |  |  |  |  |
| Hemoglobin | 105.79 (36.33) | 108.36 (42.34) | 0.065 | 110.85 (36.40) | 108.36 (42.34) | 0.063 |
| Platelets | 177 (119, 251) | 191 (136, 266) | 0.187 | 199 (146, 284) | 191 (136, 266) | 0.021 |
| WBC | 6.27 (4.18, 9.36) | 6.48 (4.52, 8.90) | 0.047 | 6.89 (4.58, 10.07) | 6.48 (4.52, 8.90) | 0.078 |
| Creatinine | 79 (61, 120) | 81 (62, 114) | 0.112 | 81 (64, 117) | 81 (62, 114) | 0.005 |
| Glucose | 7.73 (4.10) | 8.35 (3.87) | 0.156 | 8.43 (4.40) | 8.35 (3.87) | 0.019 |
| INR | 1.43 (0.78) | 1.51 (0.86) | 0.087 | 1.43 (0.75) | 1.51 (0.86) | 0.096 |

**Table S3. Patient characteristics before and after propensity score matching by treatment agent (antiplatelet only vs. non-antithrombotic)**

COPD: chronic obstructive pulmonary disease; CLD: chronic liver diseases; CHD: coronary heart disease; SBP: systolic blood pressure; SMD: standardized mean difference; WBC: white blood cell; INR: international normalized ratio

^*^ items are presented as median and quartiles.

**Table S4. Patient characteristics before and after propensity score matching by treatment agent (OAC with/without antiplatelet vs. non-antithrombotic)**

| Characteristics | Before matching | | | After matching | | |
| --- | --- | --- | --- | --- | --- | --- |
|  | Non-antithrombotic (n=4191) | OAC with/without antiplatelet (n=271) | SMD | Non-antithrombotic (n=271) | OAC with/without antiplatelet (n=271) | SMD |
| Demographics |  |  |  |  |  |  |
| Age | 63.69 (18.22) | 72.24 (15.44) | 0.507 | 71.54 (15.80) | 72.24 (15.44) | 0.045 |
| Female | 1487 (35.5) | 121 (44.6) | 0.188 | 125 (46.1) | 121 (44.6) | 0.030 |
| Smoking | 955 (22.8) | 49 (18.1) | 0.122 | 46 (17.0) | 49 (18.1) | 0.046 |
| Alcohol abuse | 722 (17.2) | 38 (14.0) | 0.100 | 33 (12.2) | 38 (14.0) | 0.074 |
| Body mass index | 22.75 (3.57) | 22.67 (3.61) | 0.022 | 22.66 (3.51) | 22.67 (3.61) | 0.004 |
| Medical history |  |  |  |  |  |  |
| Hypertension | 1577 (37.6) | 121 (44.6) | 0.143 | 124 (45.8) | 121 (44.6) | 0.022 |
| Diabetes | 1011 (24.1) | 80 (29.5) | 0.122 | 75 (27.7) | 80 (29.5) | 0.041 |
| COPD | 422 (10.1) | 43 (15.9) | 0.173 | 45 (16.6) | 43 (15.9) | 0.020 |
| CHD | 468 (11.2) | 86 (31.7) | 0.518 | 87 (32.1) | 86 (31.7) | 0.008 |
| Chronic renal failure | 542 (12.9) | 25 (9.2) | 0.118 | 25 (9.2) | 25 (9.2) | <0.001 |
| CLD | 241 (5.8) | 12 (4.4) | 0.060 | 14 (5.2) | 12 (4.4) | 0.035 |
| Malignant cancer | 572 (13.6) | 24 (8.9) | 0.152 | 21 (7.7) | 24 (8.9) | 0.040 |
| SBP | 128.52 (20.73) | 126.68 (19.67) | 0.091 | 126.98 (20.47) | 126.68 (19.67) | 0.015 |
| Laboratory events |  |  |  |  |  |  |
| Hemoglobin | 105.79 (36.33) | 112.14 (38.19) | 0.170 | 111.53 (34.00) | 112.14 (38.19) | 0.017 |
| Platelets | 177 (119, 251) | 177 (130, 251) | 0.042 | 172 (131, 245) | 177 (130, 251) | 0.046 |
| WBC | 6.27 (4.18, 9.36) | 6.02 (4.52, 9.30) | 0.075 | 6.45 (4.36, 9.97) | 6.02 (4.52, 9.30) | 0.094 |
| Creatinine | 79 (61, 120) | 84 (64, 119) | 0.294 | 80 (65, 105) | 84 (64, 119) | 0.052 |
| Glucose | 7.73 (4.10) | 7.57 (3.36) | 0.043 | 7.82 (4.14) | 7.57 (3.36) | 0.068 |
| INR | 1.43 (0.78) | 1.44 (0.73) | 0.008 | 1.48 (0.81) | 1.44 (0.73) | 0.047 |

COPD: chronic obstructive pulmonary disease; CLD: chronic liver diseases; CHD: coronary heart disease; SBP: systolic blood pressure; SMD: standardized mean difference; SD: standard deviation; WBC: white blood cell; INR: international normalized ratio

^*^ items are presented as median and quartiles.

**Table S5. Unadjusted and adjusted associations between different treatments and outcomes in patients on mechanical ventilation.**

| **Outcomes** | **Treatment regimen** | **Unadjusted OR** | **P** | **Multivariable Regression adjusted OR** | **P** | **Propensity Score Matching adjusted OR** | **P** |
| --- | --- | --- | --- | --- | --- | --- | --- |
| In-hospital mortality | Non-antithrombotic | 1 [Reference] |  | 1 [Reference] |  | 1 [Reference] |  |
|  | Antiplatelet | 1.07 (0.67, 1.69) | 0.790 | 0.81 (0.47, 1.40) | 0.448 | 0.90 (0.48, 1.69) | 0.873 |
|  | OAC with/without antiplatelet | 0.39 (0.19, 0.82) | 0.013 | 0.22 (0.08, 0.60) | 0.003 | 0.25 (0.09, 0.61) | 0.005 |
| ICU administration | Non-antithrombotic | 1 [Reference] |  | 1 [Reference] |  | 1 [Reference] |  |
|  | Antiplatelet | 0.44 (0.22, 0.86) | 0.017 | 0.38 (0.18, 0.81) | 0.013 | 0.46 (0.19, 1.04) | 0.102 |
|  | OAC with/without antiplatelet | 0.75 (0.36, 1.57) | 0.438 | 0.68 (0.27, 1.68) | 0.399 | 0.88 (0.31, 2.41) | 1.000 |
| AKI | Non-antithrombotic | 1 [Reference] |  | 1 [Reference] |  | 1 [Reference] |  |
|  | Antiplatelet | 0.51 (0.18, 1.43) | 0.200 | 0.50 (0.18, 1.39) | 0.184 | 1.00 (0.23, 4.36) | 1.000 |
|  | OAC with/without antiplatelet | 0.68 (0.21, 2.23) | 0.523 | 0.81 (0.24, 2.67) | 0.725 | 1.00 (0.18, 5.66) | 1.000 |

OAC: Oral anticoagulant; OR: odds ratio
